# Supplementary material for: Exome-Wide Association Study Identified Clusters of Pleiotropic Genetic Associations with Alzheimer’s Disease and Thirteen Cardiovascular Traits
Source: Genes (Basel). 2023 Sep 22;14(10):1834. doi: 10.3390/genes14101834 (PMC10606283; doi:10.3390/genes14101834)

Exome-Wide Association Study Identified Clusters of Pleiotropic Genetic Associations with Alzheimer's Disease and Thirteen Cardiovascular Traits

Yury Loika *, Elena Loiko, Irina Culminskaya and Alexander M. Kulminski

Biodemography of Aging Research Unit, Social Science Research Institute, Duke University, Durham, NC 27708, USA; elena.loiko@duke.edu (E.L.); irina.kulminskaya@duke.edu (I.C.); alexander.kulminski@duke.edu (A.M.K.)

* Correspondence: yury.loika@duke.edu

Supplementary Materials Listing

Supplementary materials include two Figures and three Tables:

Figure S1. Pearson's correlation coefficients between 17 considered traits, which represent cardiovascular and Alzheimer's disease risk factors in the UK Biobank.

Figure S2. Clusters of 13 traits and 13 SNPs based on Pearson's correlation coefficients as a distance measure as implemented in R function *pheatmap* from the R package *ecodist*.

Table S1. Pearson's correlation coefficients [%] between 17 considered traits, which represent cardiovascular and Alzheimer's disease risk factors in the UK Biobank.

Table S2: Univariate and pleiotropic associations of 13 SNPs in the *APOE* gene cluster and the other 4 gene loci, which demonstrated genome-wide significance of pair-wise pleiotropic associations with Alzheimer's disease and at least one of the 16 traits.

Table S3: (**A**) Patterns/clusters of pleiotropic associations of 13 SNPs in the *APOE* gene region and the other 4 gene loci, which demonstrated genome-wide significance of pair-wise pleiotropic associations with Alzheimer's disease and at least one of 16 traits. (**B**) Matrix ***A*** that was used in the cluster analysis.

Supplementary Figures

Figure S1. Pearson's correlation coefficients between 17 considered traits, which represent cardiovascular and Alzheimer's disease risk factors in the UK Biobank.

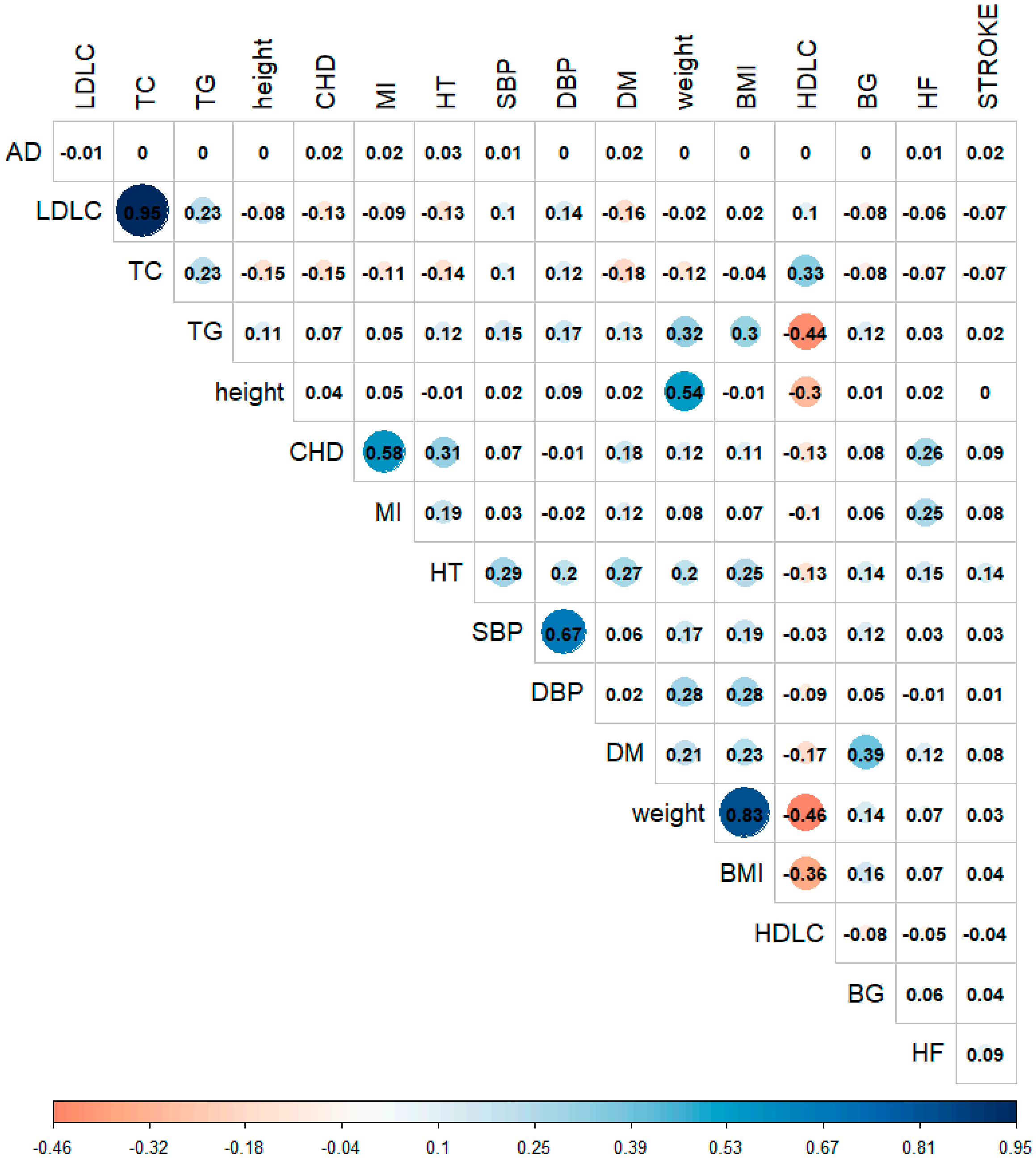

Figure S2. Clusters of 13 traits and 13 SNPs based on Pearson's correlation coefficients as a distance measure as implemented in R function *pheatmap* from the R package *ecodist*.

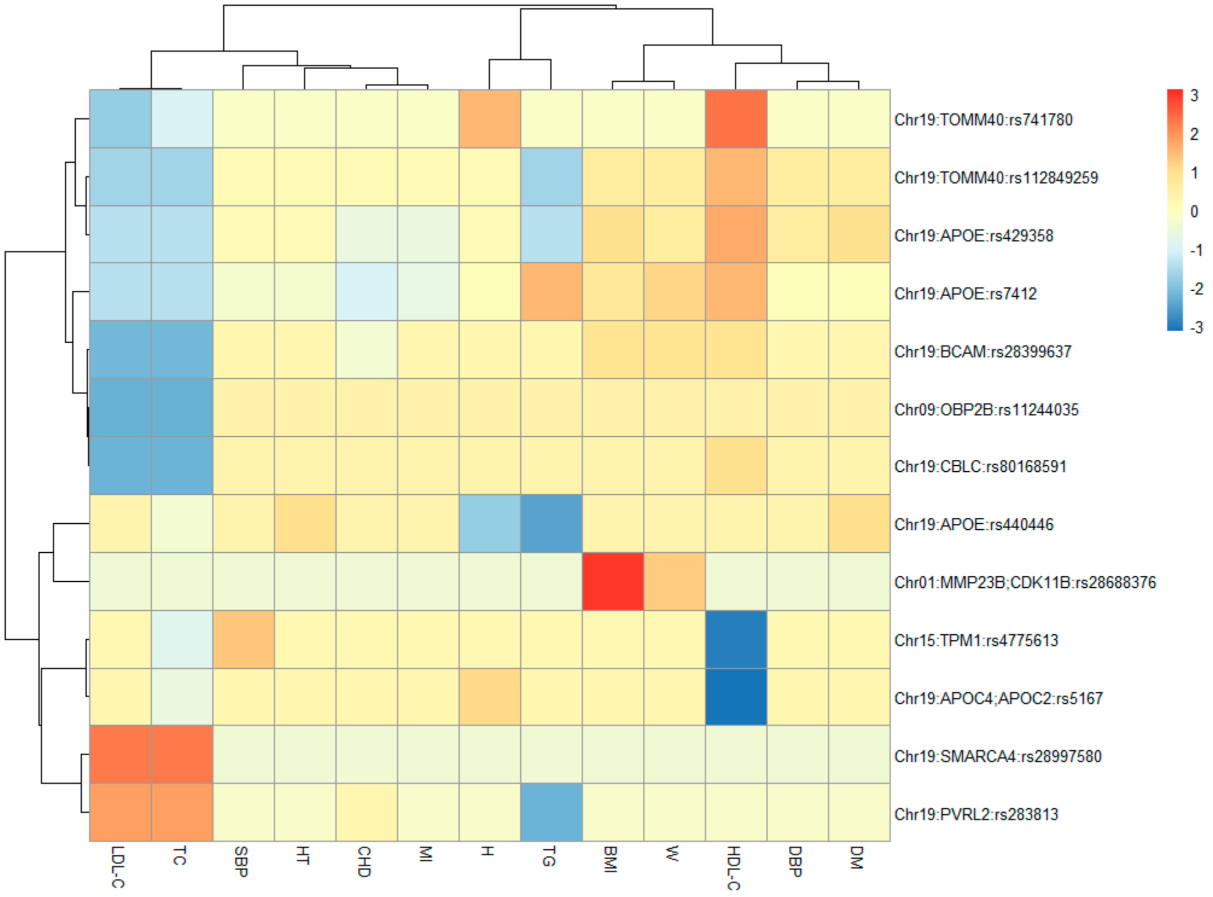

Supplement: Supplementary file 1 [file genes-14-01834-s001.zip › genes-2607992-supplementary.pdf]
